# Extensive preclinical evaluation of combined mangiferin and glycyrrhizic acid for restricting synovial neovascularization in rheumatoid arthritis

**DOI:** 10.1186/s13020-023-00863-0

**Published:** 2023-11-30

**Authors:** Xia Mao, Xiangying Yan, Congchong Li, Yudong Liu, Yanqiong Zhang, Na Lin

**Affiliations:** Research Center of Traditional Chinese Medicine Theory and Literatures, Institute of Chinese Materia Medica, China Academy of Chinese Medical Sciences, No. 16, Nanxiaojie, Dongzhimennei, Beijing, 100700 China

**Keywords:** Rheumatoid arthritis, Synovial neovascularization, Bioactive compound, Drug combination, Mangiferin, Glycyrrhizic acid

## Abstract

**Background:**

Synovial neovascularization promotes rheumatoid arthritis (RA) progression. Baihu guizhi decoction (BHGZD) has a potential in restricting this pathological change of RA.

**Purpose:**

To identify bioactive compounds (BACs) of BHGZD and to elucidate the underlying mechanisms in restricting synovial neovascularization of RA.

**Method:**

Through transcriptomic profiling, the chemical profiling of BHGZD and its effective transcriptomic profiling against RA were identified. Then, candidate targets and the corresponding BACs against synovial neovascularization were screened by “disease gene-drug target” interaction network analysis and in silico molecular docking. The binding affinities of candidate BAC-target pairs were verified using surface plasmon resonance, and the pharmacokinetic characteristics of BACs in vivo after BHGZD administration at different time points were detected by Ultra Performance Liquid Chromatography-Mass spectrum/Mass spectrum. After that, in vivo experiments based on adjuvant-induced arthritis (AIA-M) rats, and in vitro experiments based on human umbilical vein endothelial cells (HUVEC) and arthritic synovial fibroblasts (MH7A) were carried out to evaluate the pharmacological effects of BHGZD and the two-BACs-combination, and to verify the associated mechanisms.

**Result:**

VEGFA/VEGFR2/SRC/PI3K/AKT signal axis was screened as one of the key network targets of BHGZD against synovial neovascularization in RA. Mangiferin (MG) and glycyrrhizic acid (GA) were identified as the representative BACs of BHGZD for their strong binding affinities with components of the VEGFA/VEGFR2/SRC/PI3K/AKT signal axis, and their high exposed quantity in vivo. Both BHGZD and the two-BAC combination of MG and GA were demonstrated to be effective in restricting disease severity, reducing synovial inflammation and decreasing the formation of vascular opacities in AIA-M rats, and also reducing the migrative and invasive activities of HUVEC and MH7A cells and attenuating the lumen formation ability of HUVEC cells significantly. Mechanically, both BHGZD and the two-BAC combination markedly reduced the expression of VEGFA in synovial tissues, the serum levels of VEGF and NO, and the enzymatic activity of eNOS, increased the content of endostatin, and also reversed the abnormal alterations in the VEGFA/VEGFR2/SRC/PI3K/AKT signal axis in vivo and in vitro.

**Conclusion:**

MG and GA may be the representative BACs of BHGZD for restricting excessive synovial vascularization in RA via regulating VEGFA/VEGFR2/SRC/PI3K/AKT signal axis.

**Supplementary Information:**

The online version contains supplementary material available at 10.1186/s13020-023-00863-0.

## Introduction

Rheumatoid arthritis (RA) is a chronic autoimmune disease with a global prevalence of 0.5–1% [1], and its risk of occurrence in women is more than 2–3 times that of men [2], with a large number of complications, high prevalence and disability rate [3]. Commonly used drugs, such as nonsteroidal anti-inflammatory drugs (NSAIDs), biologics, glucocorticoids (GCs) and disease-modifying antirheumatic drugs (DMARDs) have been indicated to alleviate disease severity of parts of RA patients. However, growing evidence show their uncontrollable adverse effects when administrated for a long period, besides forcing financially burdensome for patients [4, 5].

Traditional Chinese medicine (TCM) has been proved to be efficacious in treating RA with unique advantages, especially in personalized treatment, due to its holism concept and syndrome differentiation characteristics [6]. Baihu-Guizhi decoction (BHGZD), a prescription of TCM from “Synopsis of Golden Chamber”, is consist of Gypsum (sulfates; Gypsum Fibrosum), *Anemarrhena asphodeloides* Bge. (Liliaceae; Anemarrhenae Rhizoma), *Cinnamomum cassia* Presl (Lauraceae; Cinnamomi Ramulus), *Oryza sativa* L. (Gramineae; Oryza Semen) and *Glycyrrhiza uralensis* Fisch (Leguminosae; Glycyrrhizae Radix et Rhizoma). It is clinically applied for relieving RA and gouty arthritis with definite efficacy [7], in particular, reaching an overall efficiency of 90%-95.45% in the treatment of RA by ameliorating clinical symptoms, such as morning stiffness, pressure pain, and swelling [8, 9].

Our previous studies reported that BHGZD may relieve RA through exerting its immunomodulatory and anti-inflammatory activities [10] and reversing energy metabolism disturbance during the disease progression [11]. Angiogenesis has been indicated to be a pivotal driver for RA, and the imbalance of pro-/anti-angiogenic factors may lead to excessive synovial vascularization, subsequently promoting inflammatory cell infiltration into the joints and exacerbating cartilage and bone destruction. Therefore, vascular neovascularization may be a promising therapeutic target for treating RA. Our previous study observed potential efficacy of BHGZD in alleviating excessive synovial vascularization of RA [12]. Herein, we aimed to identify bioactive compounds (BACs) of BHGZD and to elucidate the underlying mechanisms in restricting synovial neovascularization of RA using an integrative strategy combining transcriptional regulatory network analysis, molecular docking, surface plasmon resonance (SPR), and in vitro and in vivo experimental validations.

## Methods and materials

### Gene expression profiling and differential data analysis

To obtain the RA-related genes (differentially expressed genes (DEGs) of AIA-M model group and the normal group) and the BHGZD effective genes (DEGs of BHGZD administration group and the AIA-M model group), we downloaded the transcriptomic data of rat synovial tissue and peripheral blood cells that had been uploaded to the GEO database of NCBI by our group earlier (GSE189942. GSE190523). Significant DEGs were identified based on the criteria of P value < 0.05 and |log twofold change (FC)|> 1.0.

### Network construction and analysis

Based on ETCM 2.0 database (http://www.tcmip.cn/ETCM2/front/#/) [13], we obtained the chemical composition of BHGZD and predicted the candidate target profile referring to our previous studies [11]. The interaction information between genes in the String database (version 10.0, http://string-db.org/) [14] was used to extract the interactions between BHGZD effective genes from both transcriptomic data and TCMIP v2.0 platform, and AIA-M-related genes from transcriptomic data, and then to construct the ‘‘ disease gene-drug target ’’ interactions network. The core nodes were selected by calculating node’s degree, betweenness and closeness, and functional enrichment analysis was performed for the above core genes based on KEGG (version 96.0, https://www.kegg.jp/) [15] and Reactome database (https://reactome.org/) [16], and further hypothesized that the core pathway of BHGZD intervention in RA.

### Molecular docking stimulation

To investigate the binding abilities of two BACs in BHGZD to the target proteins, molecular docking was performed using both AutoDock Vina (The Scripps Research Institute, version 1.2.0) and Ledock (The Scripps Research Institute, version 1.0) docking software. Mol 2 files or pdb files of MG and GA were downloaded from the ZINC (version 20, http://zinc.docking.org/) [17], DrugBank (version 5.1.8, released 2021-01-03, https://www.drugbank.ca/) [18] and PubChem (last update in 2023-04-21, https://pubchem.nc-bi.nlm.nih.gov/) [19] databases, and the crystal structures of target proteins downloaded from PDB (The Protein Data Bank, https://www.rcsb.org/)[20] database. After removing the ligands and water molecules from protein structures using Discovery Studio (version 4.5) software, corresponding active pocket information of proteins molecules were predicted in PyMOL (version 2.5, https://pymol.org/2/) via GETBOX plug-in, subsequently followed by docking performed. The ligand is considered to exert strong binding affinities with receptor if the absolute value of docking score is higher than six.

### SPR detection

SPR assay was performed using Biacore 8K Biomolecular Interaction Assay System for validating the binding affinities of putative representative pharmacophores (MG and GA) to candidate targets (vascular endothelial growth factor receptor 2 antibody (VEGFR2) and proto-oncogene tyrosine-protein kinase Src antibody (SRC) in accordance with similar routine to our previous studies, with the K_D_ value less than 10^−7^ mol representing a strong binding affinity [10].

### Preparation of experimental drugs

Raw herbal materials of BHGZD were purchased from Beijing Tong Ren Tang Pharmacy, and decocted by referring to the Pharmacopoeia of the People's Republic of China (2020 edition). BACs that composed of MG (599.79 μg/kg of BHGZD, Lot No. 4773-96-0, Purity. 99.72%, Shanghai Standard Technology Co., Ltd., Shanghai, China) and GA (1185.43 μg/kg of BHGZD, Lot No. 1405-86-3, Purity. 98.00%, Shanghai Standard Technology Co., Ltd., Shanghai, China), as well methotrexate (MTX) were dissolved by dimethylsulfoxide, and diluted at 1g/mL prior to use.

### Animals

Male Lewis rats of specific-pathogen free (n = 25, 200 ± 20 g in weight, 6 ~ 8 weeks old) were purchased from Beijing Vital River Laboratory Animal Technology Co., Ltd. (Production License No. SCXK 2021-0017, Beijing, China), and housed in the Experimental Animal Center, Institute of Basic Theory for Chinese Medicine, Chinese Academy of Traditional Chinese Medicine. All experimental operations conformed to the relevant regulations on experimental animal welfare and ethics of the Ethics Committee of the Institute of Basic Theory for Chinese Medicine, Chinese Academy of Traditional Chinese Medicine (No. IBTCMCACMS21-2105-01). All animals were allowed to drink and eat freely with a constant temperature of 24 ± 1 °C and a light/dark cycle of 12 h.

### RA modeling, grouping, and treatment

A total of 25 Lewis rats were randomly divided into five groups on average: (1) normal control group; (2) adjuvant induced arthritis (AIA) -wind-hot-dampness stimulation modified model group (AIA-M); (3) AIA-M-BHGZD treatment group (21.4 g/kg, 2 times of the RA patients clinical dosage, which had been proved to exert the most prominent therapeutic effects against RA among 0.5, 1 and 2 times the daily RA patient dosage in our previous studies) [10, 11, 21]; (4) AIA-M-BACs treatment group (MG, 0.6 mg/kg; GA, 1.185 mg/kg, comparable to the corresponding content in 21.4 g/kg BHGZD); (5) AIA-MTX treatment group (0.2 mg/kg).

The AIA-M rat model was constructed in consistent with our previous studies [21], briefly, combining classical AIA model and external environmental stimulation by placing animals in the model box (production license No: RXZ-380A) for 2 h daily with certain wind velocity (6 m/s), temperature (37℃) and humidity (90%) for a period of 15 days from the day of primary immunization. Rats in normal control were simultaneously instilled with equal volume of saline solution at the same injection region. Dosing began on the day of initial immunization and continued for a total of 30 days. On the 31st day before sacrifice, rats were anesthetized by intraperitoneal injection of sodium pentobarbital, and blood was obtained from the abdominal aorta. After centrifuging, the supernatant was divided and frozen at − 80 ℃ for subsequent enzyme linked immunosorbent assay (ELISA). Bilateral ankle and knees of the hind limbs in rats were taken by removing skin and excess muscle tissues around the joints, part of which were fixed in 4% paraformaldehyde for subsequent histopathological examination, and the rest stored immediately at − 80 ℃ for western blot assay.

### Assessment of arthritis severity

The severity of arthritis in rats of different groups was evaluated by assessing arthritis prevalence, arthritis score and limb swelling using our established and proved methods [21], and please refer to the details in Additional file 1: Section S1 Severity assessment of arthritis.

### Histopathological evaluation

Samples from the hind limbs of rats that completed decalcification were removed, paraffin-embedded, cut into 4 μm slices, and stained with hematoxylin and eosin according to the general procedure, then assessed under a light microscope for synovial inflammation as well as proliferation of synovial vascular opacities in the knee joint of rats. Scoring was calculated via double-blind method [22, 23], with scores ranging from 0 to 3. Higher scores indicate more severe disease severity.

### Immunohistochemical evaluation

Paraffin sections of knee joints were taken and the expression of vascular endothelial growth factor A (VEGFA), platelet endothelial cell adhesion molecule-1 (CD31), a vascular marker in rat knee synovial tissues, was detected using Rabbit IgG immunohistochemical staining kit (Cat No. SA1028, Boster Biological Technology Co., Ltd., Wuhan, China). The sections were incubated with CD31, VEGFA antibodies at 4 °C overnight, respectively. The poly-HRP-labeled anti-rabbit IgG was used as the secondary antibody. Then, tissue sections were treated with fresh diaminobenzidine (DAB) solution until the brown positive stain developed. The integrated optical density (IOD) was measured by Image J (Image Progressing and Analysis in Java, version 1.42q, https://imagej.nih.gov/ij/) software. Detailed information of antibodies was provided in Additional file 1: Table S1 Detailed information of antibodies to CD31, VEGFA, VEGFR2, SRC, PI3K, AKT1, p-VEGFR2, p-SRC, p-PI3K, p-AKT and GAPDH proteins.

### Cell culture and treatment

Human umbilical vein endothelial cells (HUVEC, Shanghai Zishi Biotechnology Co., Ltd., Shanghai, China) and arthritis synovial fibroblasts (MH7A, Riken Cell Bank, Japan) at the generation of four to eight were chosen for in vitro experiments. Both cells were incubated in ECM medium, supplemented with 10% ~ 15% fetal bovine serum, 100 U/mL each of penicillin and streptomycin at 37 °C in a 5% CO_2_ incubator.

For drug treatment, both of HUVEC and MH7A cells were divided into seven groups as follows: (1) normal control group (isovolume medium); (2) model group (isovolume medium); (3) BHGZD treatment group (BHGZD, 7.135 μg/mL); (4) BACs treatment group (MG 0.2 ng/ mL, GA 0.4 ng/mL, as the same content of that in 7.135 μg/mL BHGZD); (5) MG treatment group (MG, 0.2 ng/mL); (6) GA treatment group (GA, 0.4 ng/mL); (7) SU5416 treatment group (SU5416, 0.238 μg/mL).

### Transwell co-culture migration assay

MH7A cells at the logarithmic growth stage were digested, resuspended in DMEM medium with 10% FBS, and inoculated in 24-well plates (600 μL, 5 × 10^4^/well). MH7A, which was seeded in advance of the plate for 24 h, was used as a model group to induce HUVEC cell migration. After 24 h of seeding, HUVEC cells at the same stage were also taken, digested. Transwell chambers were placed in the 24-well plate of advance seeding plate MH7A, and the upper chamber was added with drug and 100 μL of HUVEC cells resuspended with serum-free ECM medium (5 × 10^4^/well). After 6 h of cell migration, the chambers were fixed in 4% paraformaldehyde for 15 min, stained with 0.1% crystal violet for 15 min, washed three times with PBS, and the unmigrated cells in the upper chamber of the transwell were swabbed away with a moistened cotton swab. Cells were observed under a light microscope and photographed, and the number of cells migrating to the lower surface of the chambers was automatically analyzed using Image J software.

### Transwell migration assay

To detect the effect of BHGZD and BACs on the migration ability of HUVEC cells and MH7A cells, cells in the logarithmic growth phase were digested separately, and 100 μL of 3 × 10^4^ HUVEC cells resuspended in serum-free ECM medium, as well 100 μL of 5 × 10^4^ MH7A cells resuspended in serum-free DMEM medium. Drugs were then added to the upper chamber of the transwell. ECM medium with 15% FBS and/or a final concentration of 20 ng/mL VEGF for HUVEC cells, and DMEM medium with 10% FBS and/or a final concentration of 20 ng/mL TNF-α for MH7A cells were added to the lower chamber, respectively, followed by conventional method as in the “Transwell co-culture migration experiment”.

### Transwell invasion assay

To assay the influence of BHGZD and BACs on the invasion ability of HUVEC cells and MH7A cells, the matrix gel was melted overnight at 4 °C ahead of schedule. The bottom of the upper chamber of the transwell was wrapped with 40 μL of matrix gel per well on ice and remained for 3 h at 37 °C. HUVEC or MH7A cells containing drugs were added to the upper chamber of the transwell. ECM medium containing 20% FBS and/or a final concentration of 20 ng/mL VEGF for HUVEC cells and DMEM medium containing 20% FBS and/or a final concentration of 20 ng/mL TNF-α for MH7A cells were appended to the lower chamber, respectively. After cell invasion (20 h for HUVEC, and 24 h for MH7A), chambers were fixed in 4% paraformaldehyde for 20 min, followed by conventional method as in the “Transwell co-culture migration experiment”.

### Tube lumen formation assay

The matrix gel was melted in advance overnight at 4 °C, and 100 μL of matrix gel was added to each well of the 48-well plate on ice and then placed at 37 °C for 2 h to solidify. HUVEC cells (100 μL, 2 × 10^4^) were resuspended in ECM medium containing 5% FBS, inoculated into the 48-well plate that covered with matrix gel, and a final concentration of 20 ng/mL VEGF was added and treated for 0.5 h. After incubation in an incubator at 37 ℃ for 5 h, the cells were observed under a light microscope and photographed, and the number of branching points and branch length of the formed lumen were automatically analyzed by Image J software.

### ELISA assay

The levels of VEGF, Endostatin and nitric oxide (NO) in serum and the enzymatic activity of endothelial nitric oxide synthase (eNOS) (ml064294, ml003042, ml016840, ml003124, Shanghai Enzyme Linked Biotechnology Co., Ltd., Shanghai, China) in rats were measured using ELISA, and the regulatory effects of BHGZD, BACs and MTX on vascular neovascularization factors in rats were also investigated. In terms of MH7A cells, cells were first inoculated in 10 cm culture dishes, and 20 ng/mL TNF-α was added for reacting1 h. Cell supernatants were then collected after 24 h of drug treatment, and the levels of VEGF and NO as well as the enzymatic activity of eNOS (ml064255, ml016840, ml025095, Shanghai Enzyme Linked Biotechnology Co., Ltd., Shanghai, China) were measured to investigate the effects of BHGZD, BACs and SU5416 on the regulation of vascular neovascularization factors. Subsequently, the ratio of VEGF/ Endostatin was calculated. The absorbance was measured by Multiskan^™^ GO microplate spectrophotometer (Thermo Fisher Scientific, US).

### Western blot analysis

The expression of VEGFR2, SRC, phosphatidylinositol 3-kinase 3 antibody (PI3K), RAC-alpha serine/threonine-protein kinase antibody (AKT1), phosphorylated phospho-VEGF receptor 2(p-VEGFR2), phospho-SRC antibody (p-SRC), phospho-PI3K antibody (p-PI3K) and phospho-AKT1/2/3 antibody (p-AKT) proteins in the articular cartilage of each group of rats and HUVEC cells was detected by western blot, with glyceraldehyde-3-phosphate dehydrogenase (GAPDH) as the internal reference protein. In terms of HUVEC cells, cells were first inoculated in 10 cm culture medium, and after 24 h of drug treatment, VEGF at a final concentration of 20 ng/mL was added for 5 min for induction in all groups except the normal group, where an equal volume of medium was added. Western blot analysis was performed according to previous studies [11]. Detailed information of antibodies was provided in Additional file 1: Table S1 Detailed information of antibodies to CD31, VEGFA, VEGFR2, SRC, PI3K, AKT1, p-VEGFR2, p-SRC, p-PI3K, p-AKT and GAPDH proteins.

### Statistical analyses

GraphPad Prism (version 8.0.1, San Diego, CA, USA) software was used to calculate and analysis the experimental data, and the differences between groups were analyzed by one-way ANOVA, and the experimental data were expressed as mean ± standard deviation (*x̅* ± s). Differences were considered statistically significant when the P value was less than 0.05.

## Results

### Transcriptome data mining and network pharmacology analysis

**Fig. 1:**
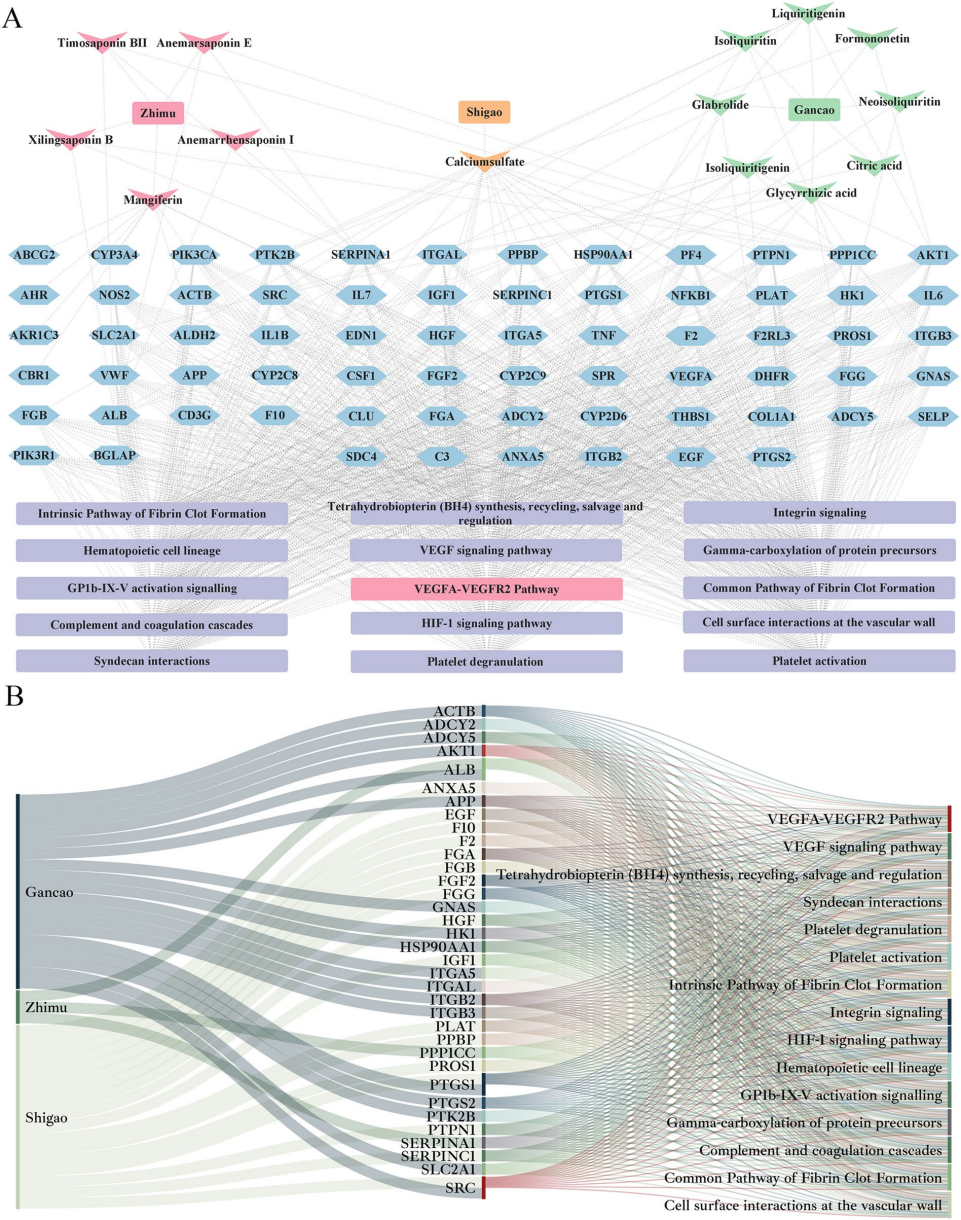
Network Illustration and Sankey diagram of three herbs contained in BHGZD (*Gypsum Fibrosum*, *Rhizoma Anemarrhenae* and *Radix Glycytthizae*), key network targets and corresponding pathways. **A** Network Illustration. The arrow nodes refer to the chemical components of the four herbs contained in BHGZD; the hexagonal nodes refer to the effective genes of BHGZD; the square nodes indicate the pathways enriched to; the size of the nodes is ordered according to their degree in the network. **B** Sankey diagram. Longer lines represent more importance of genes and pathways for BHGZD against RA

By downloading the AIA-M rat pathogenesis-related gene expression profile data from the GEO database of NCBI for our group's previous research output, 485 AIA-M-related genes were obtained by comparing the AIA-M model group with the normal group. The BHGZD candidate target profiles predicted by the TCMIP v2.0 platform were integrated with the BHGZD reverse-regulated AIA-M genes to obtain a total of 635 BHGZD effective genes. Based on the connection between AIA-M-related genes and BHGZD effective genes, a "disease-gene-drug target" interaction network was constructed, containing 659 nodes and 3614 edges; 196 core nodes were selected as candidate targets for BHGZD intervention in RA by topological feature calculation. Functional enrichment analysis was performed on the above core node genes, and we found that BHGZD key network targets were significantly involved in regulating vascular neovascularization-related pathways, such as platelet activation, complement and coagulation cascades, HIF-1 signaling pathway, hematopoietic cell lineage, VEGF signaling pathway and etc., suggesting that BHGZD may have a potential to intervene with synovial vascular neovascularization in RA. Especially, the key network targets such as SRC and AKT were significantly enriched in VEGFA-VEGFR2 signaling pathway (enrichment significance: P = 0.0198, Fig. 1).

### MG and GA may be the main BACs of BHGZD against synovial neovascularization of RA

**Fig. 2:**
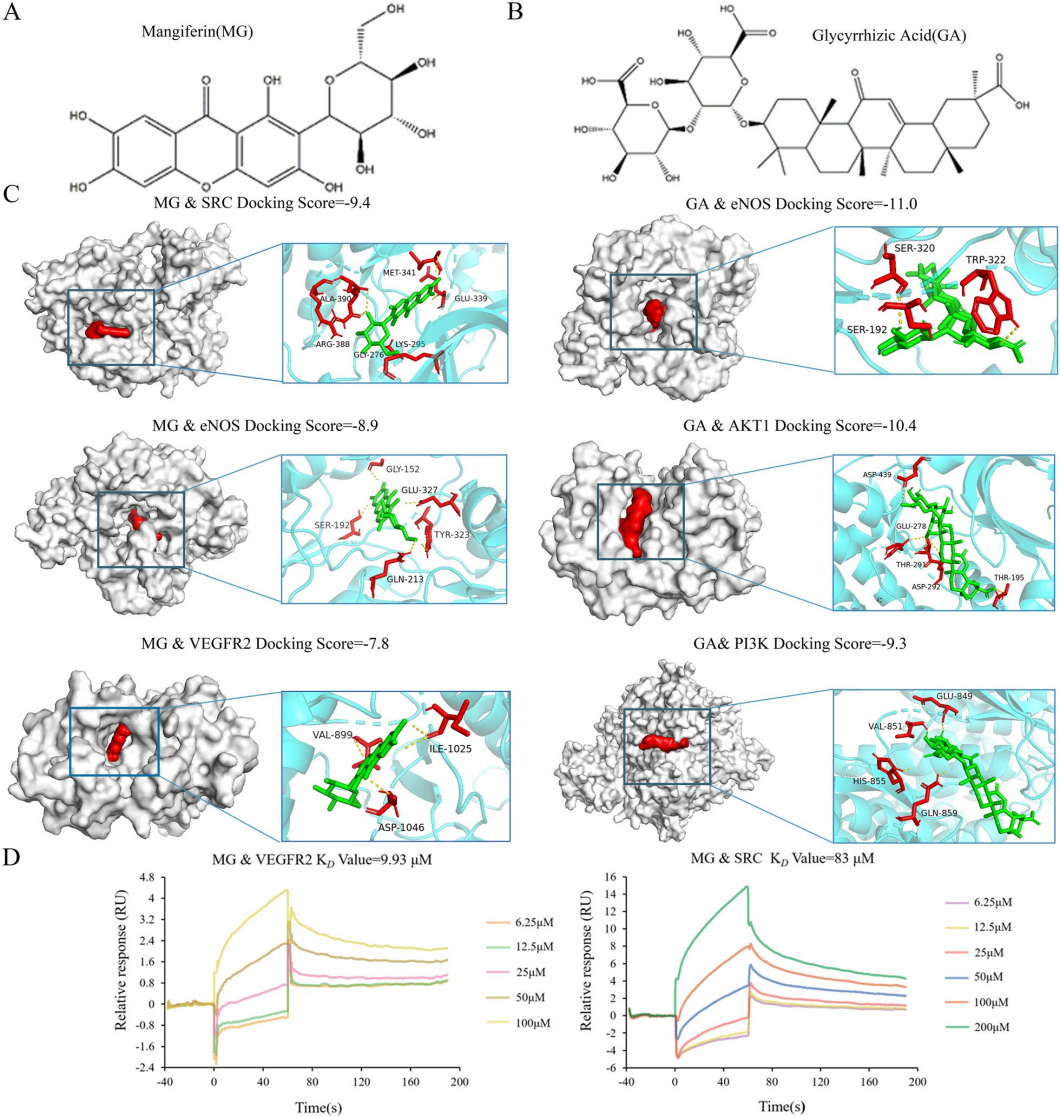
Molecular docking and SPR assay of MG and GA with the corresponding network targets involved into VEGFA-VEGFR2 signaling pathway. **A**–**B** The chemical structures of MG (left) and GA (right). **C** Molecular docking simulations of the binding patterns between MG/GA and the corresponding target proteins. **D** Binding affinity of MG with VEGFR2 or SRC verified by an SPR assay

Next, molecular docking simulations of potential BACs contained in BHGZD with key candidate targets of VEGFA-VEGFR2 signaling pathway were performed using Autodock Vina and Ledock software. The results showed several components with strong binding ability to the candidate targets, but as shown in Fig. 2C, the absolute value of all docking scores for MG and GA (chemical structures depicted in Fig. 2A, B) with SRC, eNOS, VEGFR2, PI3K, and AKT were greater than 7, so they were used as candidate components for subsequent experimental validation (Additional file 1: Table S2 Detailed information of docking parameters, the binding sites and patterns of the active components with target proteins for molecular docking of MG with SRC, eNOS, VEGFR2, as well GA with eNOS, AKT1 and PI3K). SPR technique was then applied to further confirm their strong binding affinities, with MG-VEGFR2 (K_D_ = 9.93 μM) and MG-SRC (K_D_ = 83 μM), as shown in Fig. 2D. In addition, our previous studies have examined the pharmacokinetic characteristics of MG and GA in the plasma of normal rats after BHGZD administration by Ultra Performance Liquid Chromatography-Mass spectrum/Mass spectrum (UHPLC-QTRAP-MS/MS), indicating high blood concentrations and proper absorption time [11]. The pharmacokinetic characteristics of MG and GA was described in Additional file 1: Section S2 The mean plasma concentration-time profiles of MG and GA from time 0 to 24 h after 21.4 g/kg BHGZD treatment. These data suggesting that MG and GA may serve as the main BACs of BHGZD against synovial neovascularization of RA.

### BHGZD and the two-BACs-combination of MG and GA significantly alleviate the severity of arthritis in RA rats

**Fig. 3:**
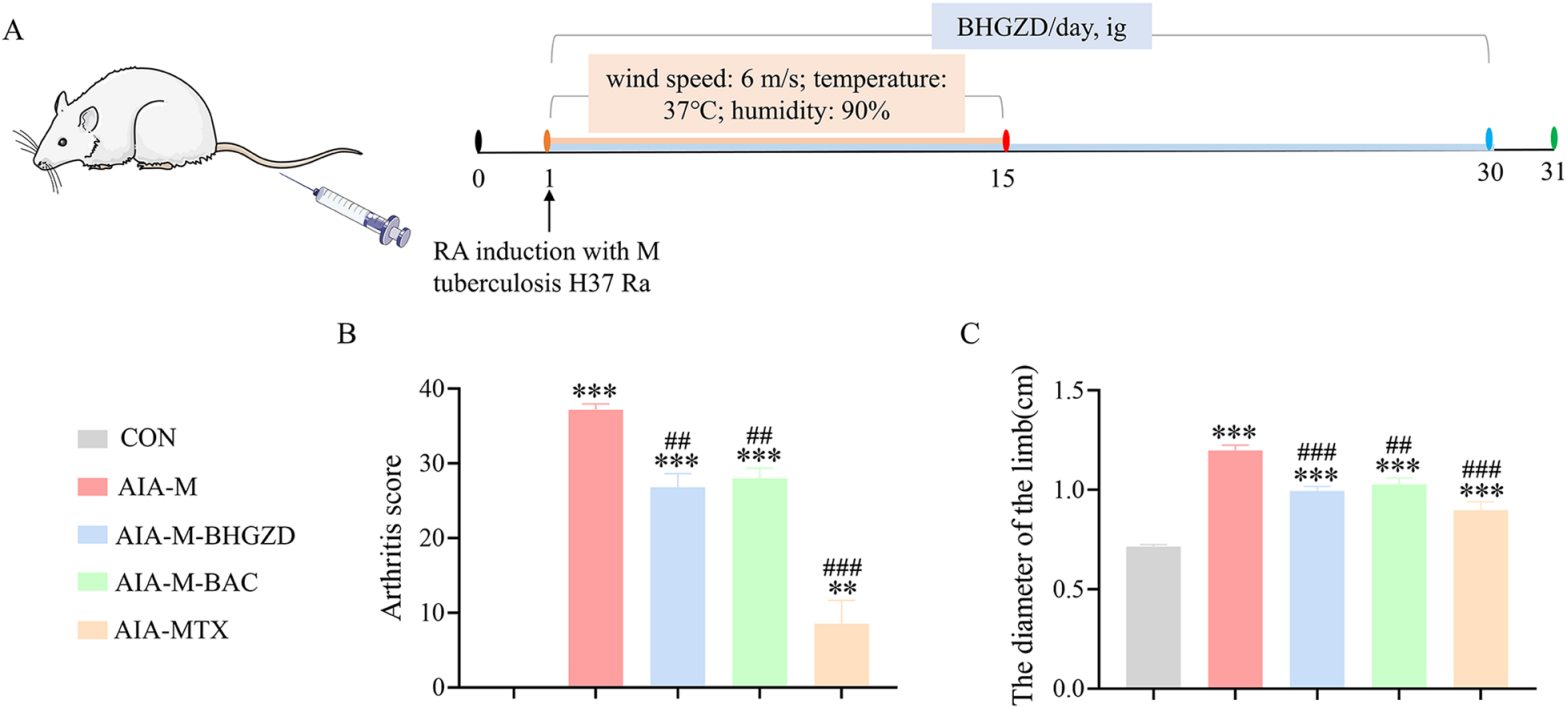
Pharmacological efficacy of BHGZD, the two-BACs-combination of MG and GA, and MTX on the severity of arthritis in AIA-M rats. **A** Arthritis induction protocol in animals. **B** Arthritis score and **C** Swelling of the right hind limb of rats in different groups (n = 5). ^*^P < 0.05, ^**^P < 0.01^***^P < 0.001, versus the normal control group; ^*#*^*P* < 0.05, ^*##*^*P* < 0.01, ^*###*^*P* < 0.001 versus the AIA-M group

**Fig. 4:**
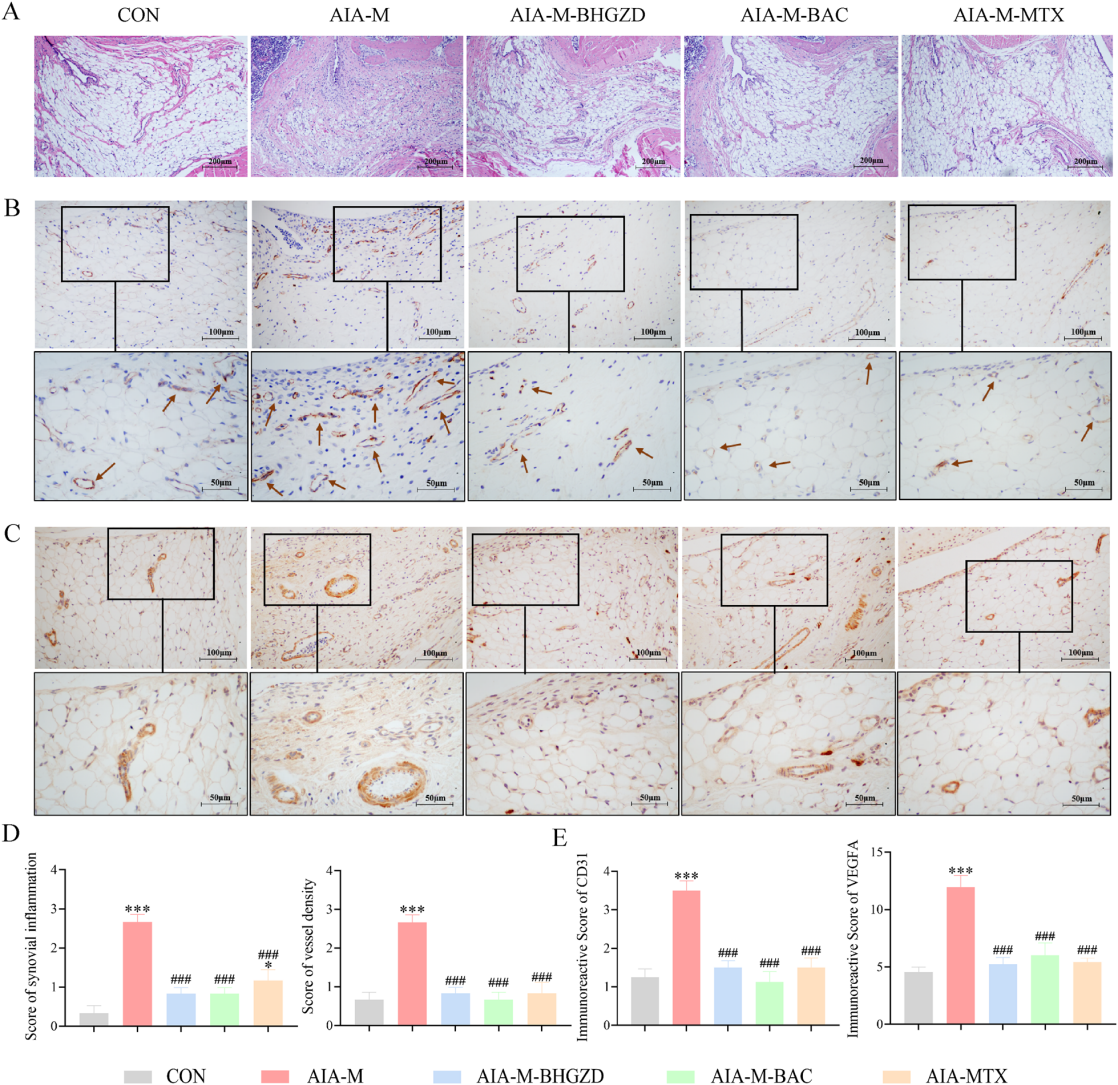
Pharmacological efficacy of BHGZD, the two-BACs-combination of MG and GA, and MTX on the angiogenesis in the synovial membrane of AIA-M rats. **A** Histopathological alterations in affected joint synovial tissues of rats in different groups assessed by HE staining (n = 6). **B**–**C** The expression of CD31 and VEGFA proteins in the synovial tissues of rats in different groups (n = 4). **D** The histopathologic scores of synovial inflammation and synovial vessel density of rats in different groups (n = 6). **E** The IOD values of CD31 and VEGFA immunohistochemical staining in the synovial tissues of rats in different groups (n = 4). Data were representative of three experiments as Mean ± SEM. ^*^P < 0.05, ^**^P < 0.01^***^P < 0.001, versus the normal control group; ^*#*^*P* < 0.05, ^*##*^*P* < 0.01, ^*###*^*P* < 0.001, versus the AIA-M group

AIA-M rat model was used to evaluate the pharmacological effects of BHGZD and the two-BACs-combination of MG and GA (Fig. 3A). We observed obvious clinical signs of joints, such as redness, swelling and deformation in rats of AIA-M group, and both the administration of BHGZD and two-BACs-combination markedly improved the degree of joint lesions, reduced clinical arthritis scores and swelling of the right hind limb in AIA-M rats (Fig. 3B** ~ C**, *P* < 0.01) (Additional file 1: Section S3 Macroscopic evidence of arthritis in different groups). According to the observation of HE staining, the synovial membrane of knee joints in AIA-M rats was abnormally hyperplastic, with a large number of inflammatory cells infiltrated, accompanied by vascular opacities formation, and displayed higher pathological score than the normal control group (Fig. 4A, D, P < 0.001). In contrast, both the administration of BHGZD and two-BACs-combination obviously inhibited the deterioration of synovial lesions in AIA-M rats by reducing synovial inflammation and vascular opacities formation (Fig. 4A, D, P < 0.001), with no statistical differences with that of MTX. Moreover, immunohistochemical staining was applied to detect the regulatory effects of BHGZD and the two-BACs-combination of MG and GA on the expression patterns of angiogenic marker CD31 and VEGFA proteins in the synovial tissues of AIA-M rats. As a result, the positive expression of CD31 (Fig. 4B) and VEGFA (Fig. 4C) proteins in the synovial membrane of the knee joint of rats in the AIA-M group was markedly increased compared with that of normal control group (*P* < 0.001, Fig. 4E), which was reversed by the administration of BHGZD, the two-BACs-combination and MTX (*P* < 0.001, Fig. 4E).

### BHGZD and the two-BACs-combination of MG and GA inhibits migration, invasion, and lumen formation in VEGF-induced HUVEC and TNF-α-induced MH7A cell models

**Fig. 5:**
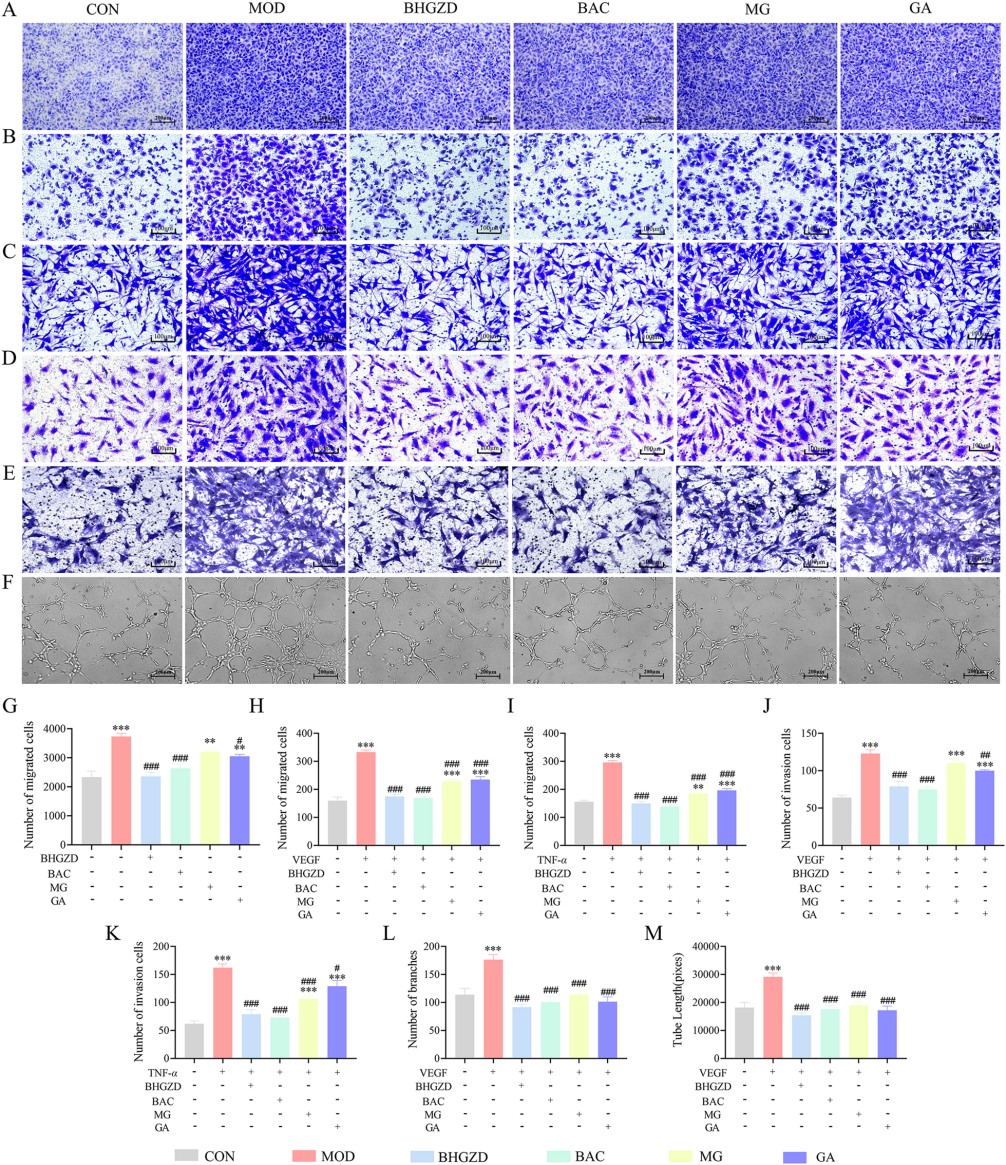
Pharmacodynamic evaluation of BHGZD and the two-BACs-combination of MG and GA in improving migrative, invasive, and lumen formation activities of VEGF-induced HUVEC and TNF-α-induced MH7A cell models. **A**, **G** Inhibitory effects of BHGZD, the two-BACs-combination or alone of MG and GA on the induction of HUVEC cell migration by co-culture of MH7A with HUVEC cells (n = 3). **B**, **H** Inhibitory effects of BHGZD, two-BACs-combination or alone of MG and GA on VEGF-induced migration of HUVEC cells (n = 5). **C**, **I** Inhibitory effects of BHGZD, two-BACs-combination or alone of MG and GA on TNF-α-induced migration of MH7A cells (n = 6). **D**, **J** Inhibitory effects of BHGZD, two-BACs-combination or alone of MG and GA on VEGF-induced HUVEC cell invasion (n = 5). **E**, **K** Inhibitory effects of BHGZD, two-BACs-combination or alone of MG and GA on TNF-α-induced invasion of MH7A cells (n = 5). **F**, **L**, **M** Inhibitory effects of BHGZD, two-BACs-combination or alone of MG and GA on VEGF-induced lumen formation in HUVEC cells (n = 5). Data were representative of three experiments as Mean ± SEM. ^***^P < 0.001, ^**^P < 0.01 versus the normal control group; ^###^*P* < 0.001, ^##^*P* < 0.01 versus the AIA-M group

To further demonstrate the involvement of BHGZD and the two-BACs-combination of MG and GA on synovial vascularization of RA, relevant cell function assays on MH7A and HUVEC cells were performed. It has been depicted evident improved migration ability of HUVEC under co-culture conditions with MH7A cells, presenting through increasing numbers of HUVEC migrating to the lower chamber in MH7A-induced group compared with the normal control group (Fig. 5A, G, P < 0.001), which was effectively reversed by the administration of BHGZD and the two-BACs-combination (Fig. 5A, G, P < 0.001). The sole application of MG and GA also slightly reduced numbers of HUVEC migration, while with no statistical difference for MG treatment. In addition, compared with normal control group, the migration numbers of HUVEC cells in the VEGF group (Fig. 5B, H, P < 0.001) as well as MH7A cells in the TNF-α group (Fig. 5C, I, P < 0.001) were significantly enhanced. BHGZD, the two-BACs-combination, MG and GA treatment dramatically inhibited the elevated migration ability of HUVEC and MH7A cells that induced by VEGF and TNF-α, respectively, among which BHGZD and the two-BACs-combination exerted superior recovery effects than MG and GA alone (Fig. 5B, C, H, I, P < 0.001). Moreover, compared with normal control group, the addition of VEGF and TNF-α significantly increased invasion extent of HUVEC (Fig. 5D, J, P < 0.001) and MH7A cells (Fig. 5E, K, P < 0.001), respectively, which was distinctly reversed by the treatment of BHGZD and the two-BACs-combination (Fig. 5D, E, J, K, P < 0.001). MG and GA reduced the invasive levels of the two cells. Meaningfully, the apparent lumen formation was observed in VEGF-induced HUVEC cells, showing increased numbers of luminal branch points and luminal branch lengths (Fig. 5F, L, M, P < 0.001). BHGZD and the two-BACs-combination markedly inhibited the lumen formation ability of HUVEC cells induced by VEGF, with better effects than that of MG and GA treatment alone (Fig. 5F, L, M, P < 0.001).

### *BHGZD and the two-BACs-combination of MG and GA reverses the abnormal expression of angiogenic factors *in vivo* and *in vitro

**Fig. 6:**
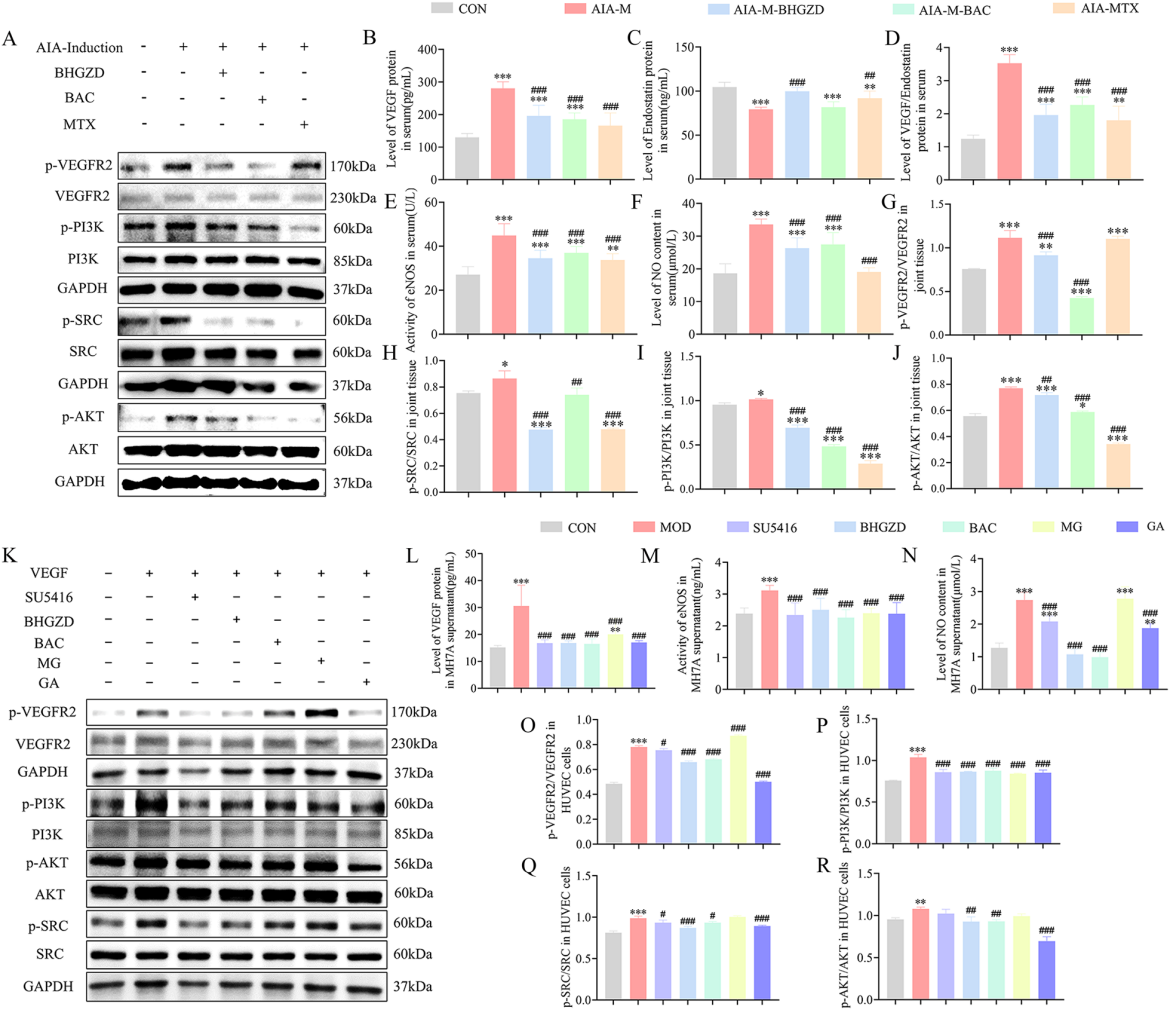
Regulatory effects of BHGZD and two-BAC-combination on the expression of corresponding target proteins involved into the VEGFA/VEGFR2/SRC/PI3K/AKT signal axis in AIA-M rat joints and MH7A and HUVEC cells. **A**, **G** ~ **J** The expression levels of p-VEGFR2, VEGFR2, p-PI3K, PI3K, p-SRC, SRC, p-AKT and AKT in AIA-M rat joints in different groups detected by western blot analysis (n = 3). **B** ~ **F** The levels of VEGF, Endostatin and NO in serum and the enzymatic activity of eNOS in rats detected by ELISA (n = 6). **K**, **O** ~ **R** The protein expression of p-VEGFR2, VEGFR2, p-PI3K, PI3K, p-SRC, SRC, p-AKT and AKT of VEGF-induced HUVEC cells in different groups detected by western blot analysis (n = 3). **L** ~ **N** The levels of VEGF, NO and the enzymatic activity of eNOS in the cell supernatant of TNF-α-induced MH7A cells in different groups detected by ELISA. (n = 6). Data were representative of three experiments as Mean ± SEM. ^***^P < 0.001, ^**^P < 0.01 versus the control group; ^###^*P* < 0.001, ^##^*P* < 0.01 versus the AIA-M group

As have been well recognized that VEGF and endostatin are of the most potent angiogenic and inhibitory factors, the balance of which maintain the homeostatic state of in vivo angiogenesis, and subsequently influence the pathological process of RA. We found that the ratio of VEGF/endostatin was abnormally elevated in rats of AIA-M group, which was significantly reversed by the treatment of BHGZD, the two-BACs-combination and MTX (Fig. 6B ~ D, *P* < 0.01). The similar trend was observed in TNF-α induced MH7A cells (Fig. 6L, P < 0.001).

### BHGZD and the two-BACs-combination of MG and GA ameliorates the disease severity of RA by regulating the VEGF/VEGFR2/SRC/PI3K/AKT signal axis

ELISA and western blot analysis were performed to determine the regulatory effects of BHGZD and the two-BACs-combination of MG and GA on the corresponding targets involved into the VEGFA/VEGFR2/SRC/PI3K/AKT signal axis. As shown in Fig. 6A, G ~ J, the ratios of p-VEGFR2/VEGFR2, p-SRC/SRC, p-PI3K/PI3K and p-AKT/AKT in the joint tissues of rats in the AIA-M group were markedly higher than those in the normal control group (*P* < 0.05), which were dramatically reversed by the treatment of BHGZD, the two-BACs-combination and MTX (*P* < 0.01). The same trends were observed in VEGF-induced HUVEC cells by particularly adding VEGFR2 tyrosine kinase inhibitors named SU5416 (Fig. 6K, O ~ R, *P* < 0.05). Moreover, both NO content and eNOS enzyme activity were markedly increased in serum of AIA-M rats compared with the normal control group (Fig. 6E ~ F, *P* < 0.001). Notably, the administration of BHGZD, the two-BACs-combination and MTX significantly reduced these changes to nearly normal levels (Fig. 6E ~ F**,** *P* < 0.001), in line with the findings observed based on the TNF-α-induced MH7A cells (Fig. 6M** ~ **N**,** *P* < 0.001).

## Discussion

Growing evidence indicates that synovial vascularization may play an essential role in promoting the aggressive progression of RA [24]. The inflammatory microenvironment in synovial tissues and excessive activation of platelets disrupt the balance of angiogenic regulators in the body, and thus induces excessive synovial vascularization, leading to the formation of vascular opacities, that are of initiating factors for joint pathology and cartilage destruction [25]. Our previous studies reported prominent effects of BHGZD in restricting synovial hypervascularization in RA, however, with main BACs and corresponding acting mechanisms unclarified. It has been well accepted that BACs extracted from herbal prescriptions may be promising drug candidates for the treatment of diseases and also an excellent source of drug discovery [26]. Therefore, in the current study, a systematic approach combining molecular docking, SPR and pharmacokinetic assays was carried out to identify MG and GA as the candidate BACs of BHGZD in restricting excessive synovial vascularization during RA progression. Interestingly, both MG and GA have been reported to have vascular protective efficacy through inhibiting neovascularization and reducing synovial inflammation.

To confirm whether BHGZD and the two-BACs-combination of MG and GA alleviated RA through inhibiting the revascularization process, the expression of CD31 and VEGFA proteins, both of which act as indicators for active intra-synovial revascularization [27], were detected. Our data showed that both the administration of BHGZD and the two-BACs-combination of MG and GA dramatically decreased the positive expression of CD31 and VEGFA proteins in the synovial tissues of knee joints in AIA-M rats. Accumulating studies reported that the development of angiogenesis may depend on the proliferation, migration, invasion and lumen formation of HUVEC cells in the intravascular layer [28]. Consistently, we herein found that BHGZD and the two-BACs-combination not only weakened the migrative and invasive abilities of VEGF-induced HUVEC cells, TNF-α-induced MH7A cells and co-cultured MH7A-induced HUVEC cells, but significantly inhibited the lumen formation ability of VEGF-induced HUVEC cells.

**Fig. 7:**
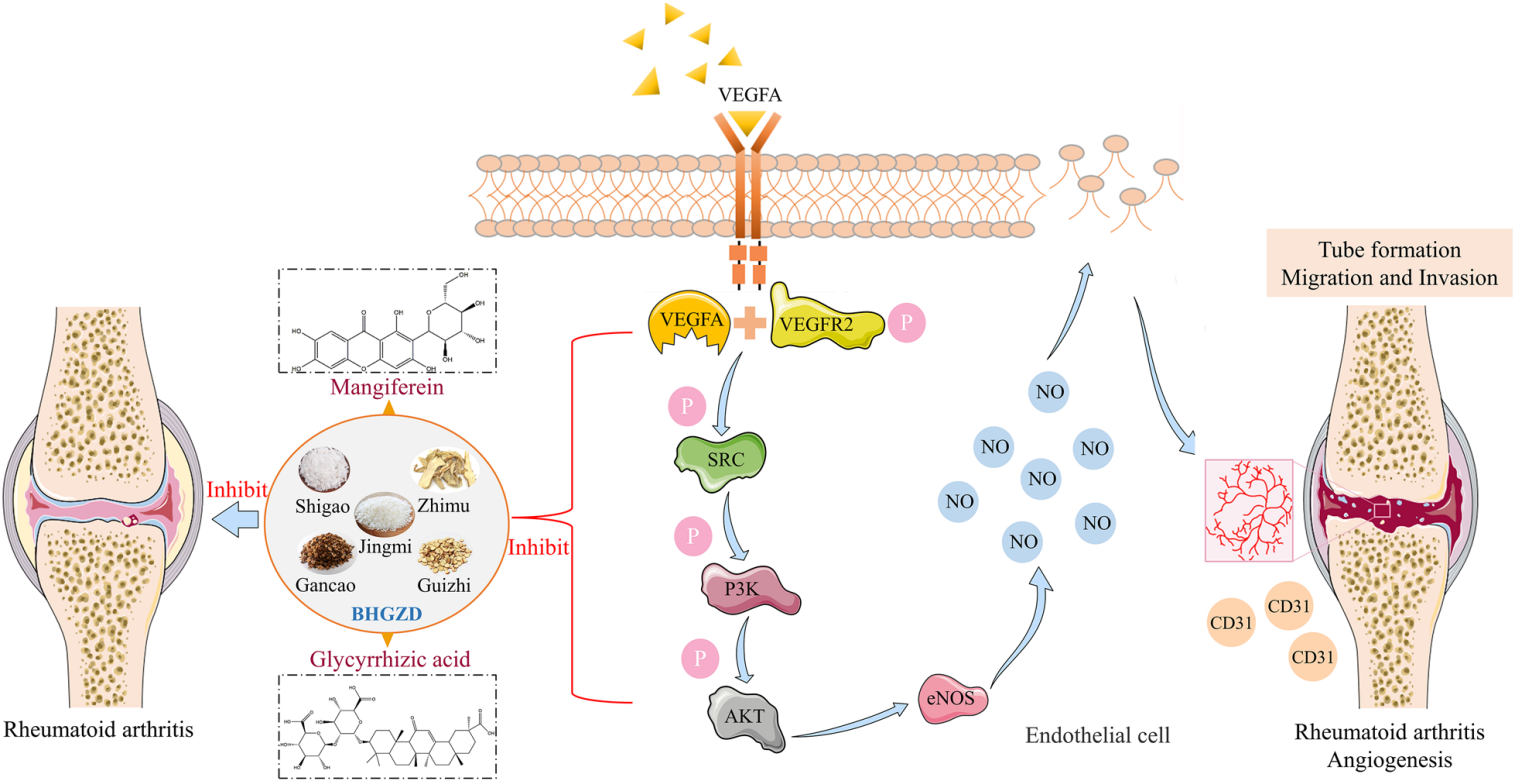
Schematic depiction of the underlying mechanism of BHGZD and the two-BACs-combination of MG and GA restricting synovial neovascularization in RA via the VEGFA/VEGFR2/SRC/PI3K/AKT signaling axis

Network analysis of the transcriptomic profiling of the whole blood cells and synovium tissues collected from rats in normal control, AIA-M model and AIA-M-BHGZD treatment groups indicated VEGFA/VEGFR2/PI3K/AKT can be a possible pathway of BHGZD intervening with RA. VEGF, a factor of endothelial cell growth/survival, serve as a key mediator of vascular neogenesis [29]. Although there are several related genes including VEGFB, VEGFC, and placental growth factor, most attention is focused on VEGFA due to its up-stream key role in regulating angiogenesis during homeostasis and disease [30]. The activation of signals through the VEGF receptor class IV tyrosine kinase receptor family by binding to the VEGFR2 receptor has been indicated to promote vascular endothelial cell mitogenesis and permeability [30, 31]. It regulates SRC activity and revascularization-related molecules, leading to the increased vascular permeability, as well proliferation and migration of endothelial cells [32]. Moreover, PI3K/AKT signaling, as an important downstream component of VEGFA-VEGFR2-SRC signaling, has been indicated to be closely related to RA synovial vascularization [33, 34]. PI3K can be activated by VEGFR2-SRC axis, subsequently causes AKT phosphorylation, and promotes vascular renewal progress [35]. Especially, as one of the important substrates of AKT, released NO by eNOS can enhance the migration and homing of endothelial cells, and promote vascular neogenesis [36, 37]. Our in vivo experiments showed that the expression of p-VEGFR2 protein was significantly elevated in the joint tissues of AIA-M rats, accompanied by an increasing expression of p-SRC, p-PI3K and p-AKT proteins, which were all markedly reversed to a nearly normal level by the treatment of BHGZD and the two-BACs-combination of MG and GA. Moreover, SU5416, a highly selective VEGFR2 tyrosine kinase inhibitor [38, 39], was used to inhibit the phosphorylation of VEGFR2 in vitro experiments. All the SU5416, BHGZD and the two-BACs-combination interventions significantly decreased VEGF and NO content, and the enzymatic activity of eNOS in the supernatant of MH7A cells, and reduced the ratios of p-VEGFR2/VEGFR2, p-SRC/SRC, p-PI3K/PI3K and p-AKT/AKT in VEGF-induced HUVEC cells, all of which suggesting that both BHGZD and the two-BACs-combination may inhibit synovial vascularization in RA by interfering with the VEGFA/VEGFR2/SRC/PI3K/AKT signal axis (Fig. 7).

Compared with modern medicine, BHGZD have the beneficial properties of TCM in preventing the emergence of resistance and increasing therapeutic effects with seldom adverse effects in clinical practice. Accumulating studies have revealed that only a few BACs may be responsible for clinical effects of herbal formulae, with advantages of definite composite components, easy availability of drugs, and clarified pharmacological mechanisms. Therefore, the identification of BACs contained in herbs or herbal formulae has been strongly recommended [40]. Interestingly, we herein demonstrated that the two-BACs-combination of MG and GA may significantly inhibit the excessive synovial vascularization in RA and have enormous potentials in the development of novel drugs for the therapeutics of this disease. In the future, a series of further scientific research, such as pharmaceutical identification, preclinical and clinical evaluation on therapeutic effects and safety are demanded to promote the clinical transformation and application of this two-BACs-combination.

## Conclusion

The current study offer an evidence that MG and GA may be the main BACs of BHGZD for their strong binding affinities with components of predicted targeted signal axis, high exposed quantity in vivo, and prominent effects in restricting disease severity by reducing synovial inflammation and restricting excessive synovial vascularization during RA progression, the mechanisms of which may be associated with its regulatory effects on the VEGFA/VEGFR2/SRC/PI3K/AKT signal axis. These findings provide a promising candidate drug combination for RA therapy, that shed light into new drug discovery for targeting synovial neovascularization in RA, and also promote modernization of TCM.

### Supplementary Information

**Additional file 1: Table S1.** Detailed information of antibodies to CD31, VEGFA, VEGFR2, SRC, PI3K, AKT1, p-VEGFR2, p-SRC, p-PI3K, p-AKT and GAPDH proteins. **Table S2.** Detailed information of docking parameters, the binding sites and patterns of the active components with target proteins for molecular docking of MG with SRC, eNOS, VEGFR2, as well GA with eNOS, AKT1 and PI3K. **Section S1.** Severity assessment of arthritis. **Section S2.** The mean plasma concentration-time profiles of MG and GA from time 0 to 24 h after 21.4 g/kg BHGZD treatment. **Section S3.** Macroscopic evidence of arthritis in different groups.

## Data Availability

All data generated or analyzed during this study are included in this published article and its Additional file.

## Abbreviations

AIA: Adjuvant induced arthritis
AIA-M: AIA-wind-hot-dampness stimulation modified model group
AKT1: RAC-alpha serine/threonine-protein kinase
BACs: Bioactive compounds
BHGZD: Baihu guizhi decoction
CD31: Platelet endothelial cell adhesion molecule-1
DAB: Diaminobenzidine
DEGs: Differentially expressed genes
DMARDs: Disease-modifying antirheumatic drugs
ELISA: Enzyme linked immunosorbent assay
Enos: Endothelial nitric oxide synthase
GA: Glycyrrhizic acid
GAPDH: Glyceraldehyde-3-phosphate dehydrogenase
GCs: Glucocorticoids
HUVEC: Human umbilical vein endothelial cells
IOD: Integrated optical density
MG: Mangiferin
MH7A: Arthritic synovial fibroblasts
MTX: Methotrexate
NO: Nitric oxide
NSAIDs: Nonsteroidal anti-inflammatory drugs
p-AKT: Phospho-AKT1/2/3
PI3K: Phosphatidylinositol 3-kinase 3
p-PI3K: Phospho-PI3K
p-SRC: Phospho-SRC
p-VEGFR2: Phosphorylated phospho-VEGF receptor 2
RA: Rheumatoid arthritis
SPR: Surface plasmon resonance
SRC: Proto-oncogene tyrosine-protein kinase Src
TCM: Traditional Chinese medicine
UHPLC-QTRAP-MS/MS: Ultra performance liquid chromatography-mass spectrum/mass spectrum
VEGFA: Vascular endothelial growth factor A
VEGFR2: Vascular endothelial growth factor receptor 2
