# Supplementary material for: Extensive preclinical evaluation of combined mangiferin and glycyrrhizic acid for restricting synovial neovascularization in rheumatoid arthritis
Source: Chin Med. 2023 Nov 30;18:156. doi: 10.1186/s13020-023-00863-0 (PMC10687849; doi:10.1186/s13020-023-00863-0)
Supplement: Supplementary file 1 — Additional file 1: Table S1. Detailed information of antibodies to CD31, VEGFA, VEGFR2, SRC, PI3K, AKT1, p-VEGFR2, p-SRC, p-PI3K, p-AKT and GAPDH proteins. Table S2. Detailed information of docking parameters, the binding sites and patterns of the active components with target proteins for molecular docking of MG with SRC, eNOS, VEGFR2, as well GA with eNOS, AKT1 and PI3K. Section S1. Severity assessment of arthritis. Section S2. The mean plasma concentration-time profiles of MG and GA from time 0 to 24 h after 21.4 g/kg BHGZD treatment. Section S3. Macroscopic evidence of arthritis in different groups. [file 13020_2023_863_MOESM1_ESM.docx]

Additional file

**List of** Additional file

Additional file 1: Table S1. Detailed information of antibodies to CD31, VEGFA, VEGFR2, SRC, PI3K, AKT1, p-VEGFR2, p-SRC, p-PI3K, p-AKT and GAPDH proteins

Additional file 1: Table S2. Detailed information of docking parameters, the binding sites and patterns of the active components with target proteins for molecular docking of MG with SRC, eNOS, VEGFR2, as well GA with eNOS, AKT1 and PI3K

Section 1. Severity assessment of arthritis

Section 2. The mean plasma concentration-time profiles of MG and GA from time 0 to 24 h after 21.4 g/kg BHGZD treatment

Section 3. Macroscopic evidence of arthritis in different groups

Additional file 1: **Table S1. Detailed information of antibodies to CD31, VEGFA, VEGFR2, SRC, PI3K, AKT1, p-VEGFR2, p-SRC, p-PI3K, p-AKT and GAPDH proteins**

| **Antibodies** | **Molecular weight** | **Cat No.** | **Sources** | **Concentration** | **Companies** |
| --- | --- | --- | --- | --- | --- |
| Platelet endothelial cell adhesion molecule | 82kDa | ab182981 | rabbit monoclonal antibody | 1:1000 | Abcam  Cambridge, UK |
| Vascular endothelial growth factor A | 27kDa | A12303 | rabbit polyclonal antibody | 1:100 | Abclonal  Wuhan, China |
| Vascular endothelial growth factor receptor 2 | 230kDa | 9698 | rabbit monoclonal antibody | 1:2000 | Cell Signaling Technology  Boston, USA |
| Proto-oncogene tyrosine-protein kinase Src | 60kDa | 2109 | rabbit monoclonal antibody | 1:2000 | Cell Signaling Technology  Boston, USA |
| Phosphatidylinositol 3-kinase 3 | 85kDa | 4257 | rabbit monoclonal antibody | 1:2000 | Cell Signaling Technology  Boston, USA |
| RAC-alpha serine/threonine-protein kinase | 60kDa | ab89402 | mouse monoclonal antibody | 1:2000 | Abcam  Cambridge, UK |
| Phosphorylated phospho-VEGF receptor 2 | 170kDa | AF3279 | rabbit polyclonal antibody | 1:2000 | Affinity Biosciences  OH, USA |
| Phospho-SRC antibody | 60kDa | AF3161 | rabbit polyclonal antibody | 1:2000 | Affinity Biosciences  OH, USA |
| Phospho-PI3K antibody | 56kDa | AF3242 | rabbit polyclonal antibody | 1:2000 | Affinity Biosciences  OH, USA |
| Phospho-AKT1/2/3 | 56kDa | AF0016 | rabbit polyclonal antibody | 1:2000 | Affinity Biosciences  OH, USA |
| Glyceraldehyde-3-phosphate dehydrogenase | 37kDa | 5174T | rabbit monoclonal antibody | 1:10000 | Cell Signaling Technology  Boston, USA |

Additional file 1: **Table S2. Detailed information of docking parameters, the binding sites and patterns of the active components with target proteins for molecular docking of MG with SRC, eNOS, VEGFR2, as well GA with eNOS, AKT1 and PI3K**

| **Target protein** | **Bioactive components** | **Combining the center coordinates of**  **the cavity (X, Y, Z)** | **Binding sites** | **Hydrogen-binding** |
| --- | --- | --- | --- | --- |
| eNOS | MG | 14.8, 14.2, 57.4 | GLY152, GLU327, TYR323, GLN213, SER192 | \ |
| SRC | MG | 20.7, 20.0, 57.7 | MET341, LYS295, ARG388, ALA390, GLY276, GLU339 | MET341, LYS295, ARG388 |
| VEGFR2 | MG | 15.2, -1.9, 9.7 | ASP1046, VAL899, ILE1025 | ASP1046 |
| eNOS | GA | 14.8, 14.2, 57.4 | SER320, TRP322, SER192 | \ |
| AKT1 | GA | -7.5, 1.9, 17.6 | ASP439, GLU278, THR291, ASP292, THR195 | ASP-439 |
| PI3K | GA | -19.2, 11.5, 28.0 | GLU849, VAL851, HIS855, GLN859 | \ |

Additional file 1: *Section 1.* *Severity assessment of arthritis*

Rats in different groups were observed once every day after primary immunization. Arthritis severity was evaluated by arthritis prevalence, arthritis score and limb swelling, which were measured by 2 independent, blinded observers up to the day before sacrifice.

(1) Arthritis prevalence was the number of arthritis rats divided by total number of rats in each group.

(2) The arthritis score was the total of the scores for all 4 limbs (maximum possible arthritis score 40).

(3) Hind paw thickness was measured by vernier caliper.

Additional file 1: *Section 2. The mean plasma concentration-time profiles of MG and GA from time 0 to 24 h after 21.4 g/kg BHGZD treatment*

In our previous study (Li *et al*., 2021), we determined the pharmacokinetic characteristics of Mangiferin (MG) and Glycyrrhizic Acid (GA) in the plasma of normal rats at different time points after BHGZD administration by UHPLC-QTRAP-MS/MS. As shown in **Supplementary Figure 1**, the time to reach the maximum blood concentration of MG and GA within 24 h of gavage was 4 h and 4.7 h, respectively, and the maximum blood concentrations were 132.006 μg/L and 46.466 μg/L, respectively. The results showed that both MG and GA could be introduced into the blood, and there was a certain distribution of blood concentration in rats.


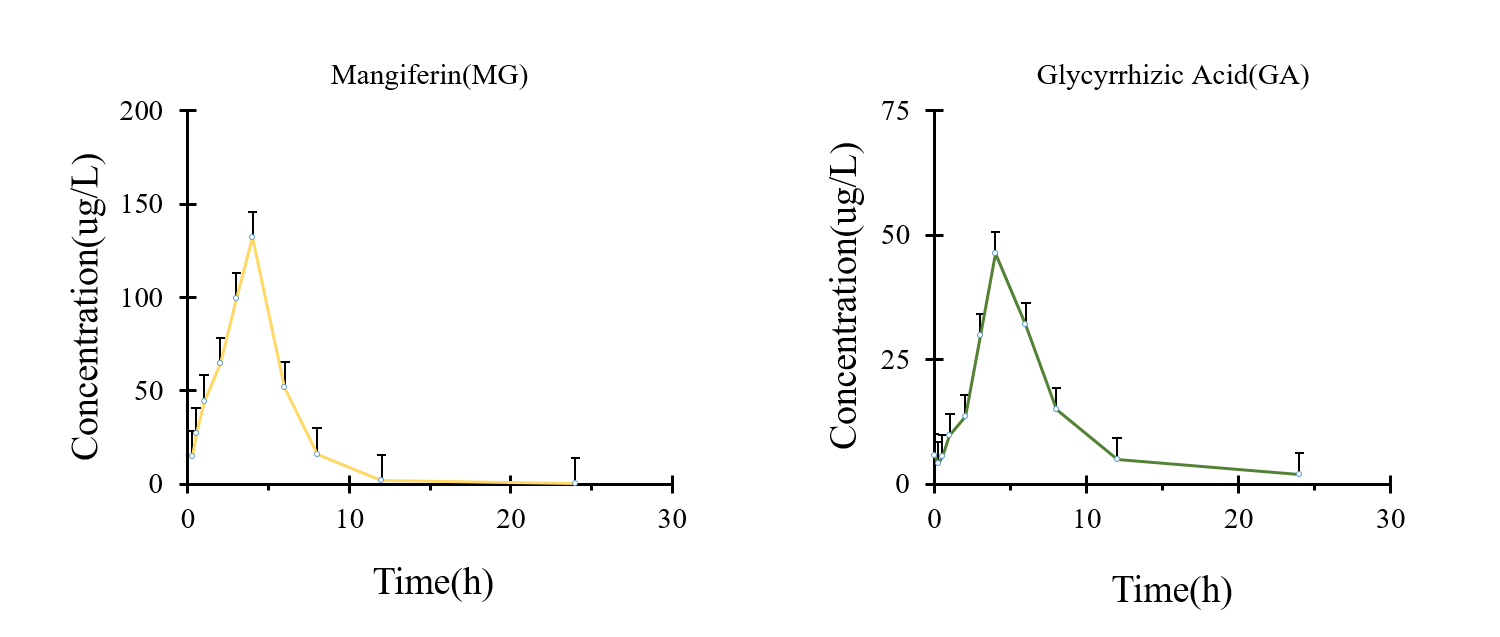


Additional file 1: **Figure 1** The mean plasma concentration-time profiles of MG and GA from time 0 to 24 h after 21.4 g/kg BHGZD treatment (n = 3).

Additional file 1: *Section 3. Macroscopic evidence of arthritis in different groups*

In our previous study (Mao *et al*., 2022), based on the AIA-M rat model, we observed significant clinical signs, such as erythema and deformity, in the joints of rats in the AIA-M group, whereas the administration of both BHGZD and the two BACs-combination significantly improved the degree of arthropathy and reduced clinical arthritis scores and swelling in the right hind limb of the AIA-M rats (Additional file 1: **Figure 2**). The arthritis and right hind limb swelling scores for evaluating the efficacy of BHGZD and BACs to improve arthritis in AIA-M rats can be seen in **Figure 3B~C** in this manuscript.


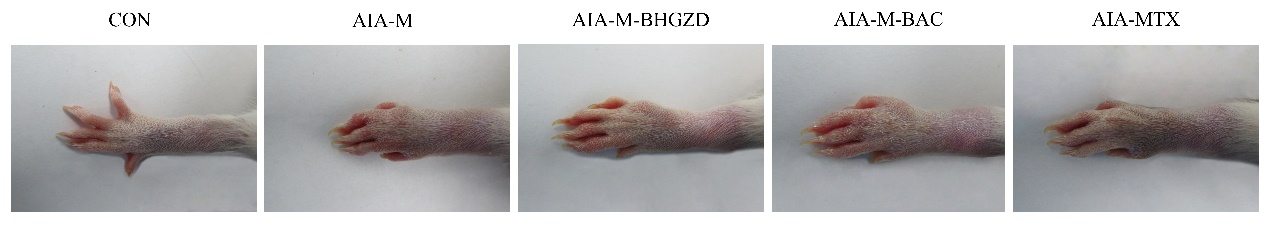


Additional file 1: **Figure 2** Macroscopic evidence of arthritis in different groups.

**References**

Li, W., Mao, X., Wang, X., Liu, Y., Wang, K., Li, C., Li, T., Zhang, Y., Lin, N., 2021. Disease-Modifying Anti-rheumatic Drug Prescription Baihu-Guizhi Decoction Attenuates Rheumatoid Arthritis via Suppressing Toll-Like Receptor 4-mediated NLRP3 Inflammasome Activation. Front Pharmacol. 5;12:743086. doi: 10.3389/fphar.2021.743086.

Mao, X., Liu, Y., Li, W., Wang, K., Li, C., Wang, Q., Chen, W., Ma, Z., Wang, X., Ding, Z., Zhang, Y., Lin, N. 2022. A promising drug combination of mangiferin and glycyrrhizic acid ameliorates disease severity of rheumatoid arthritis by reversing the disturbance of thermogenesis and energy metabolism. Phytomedicine. 104:154216. doi:10.1016/j.phymed.2022.154216
